# Renal Injuries after Cardiac Arrest: A Morphological Ultrastructural Study

**DOI:** 10.3390/ijms23116147

**Published:** 2022-05-30

**Authors:** Maria Tsivilika, Dimitrios Kavvadas, Sofia Karachrysafi, Katerina Kotzampassi, Vasilis Grosomanidis, Eleni Doumaki, Soultana Meditskou, Antonia Sioga, Theodora Papamitsou

**Affiliations:** 1 Laboratory of Histology-Embryology, School of Medicine, Faculty of Health, Aristotle University of Thessaloniki, 54124 Thessaloniki, Greece; tsivilikamaria@gmail.com (M.T.); kavvadas@auth.gr (D.K.); sofia_karachrysafi@outlook.com (S.K.); sefthym@auth.gr (S.M.); sioga@auth.gr (A.S.); 2 Department of Surgery, Aristotle University of Thessaloniki, AHEPA Hospital, 54636 Thessaloniki, Greece; kakothe@yahoo.com; 3 Department of Anesthesiology and ICU, Aristotle University Thessaloniki, 54124 Thessaloniki, Greece; grosoman@otenet.gr; 4 1st Department of Internal Medicine, Faculty of Medicine, AHEPA University Hospital, Aristotle University of Thessaloniki, 54636 Thessaloniki, Greece; elenidoumaki@yahoo.gr

**Keywords:** kidneys, cardiac arrest, renal injury, lesions, electron microscopy

## Abstract

Background: This study aims to investigate the probable lesions and injuries induced in the renal tissue after a cardiac arrest. The renal ischemia–reperfusion model in cardiac arrest describes the effects of ischemia in the kidneys, alongside a whole-body ischemia–reperfusion injury. This protocol excludes ischemic conditions caused by surgical vascular manipulation, venous injury or venous congestion. Methods: For the experimental study, 24 swine were subjected to cardiac arrest. Seven minutes later, the cardiopulmonary resuscitation technique was performed for 5 min. Afterwards, advanced life support was provided. The resuscitated swine consisted one group and the non-resuscitated the other. Tissue samples were obtained from both groups for light and electron microscopy evaluation. Results: Tissue lesions were observed in the tubules, parallel to destruction of the microvilli, reduction in the basal membrane invaginations, enlarged mitochondria, cellular vacuolization, cellular apoptosis and disorganization. In addition, fusion of the podocytes, destruction of the Bowman’s capsule parietal epithelium and abnormal peripheral urinary space was observed. The damage appeared more extensive in the non-resuscitated swine group. Conclusions: Acute kidney injury is not the leading cause of death after cardiac arrest. However, evidence suggests that the kidney damage after a cardiac arrest should be highly considered in the prognosis of the patients’ health outcome.

## 1. Introduction

Ischemia of the whole body caused by cardiac arrest (CA) has many adverse outcomes and, even after the restoration of the systemic circulation, can lead to death or disability, mainly due to irreversible brain injury [1]. Τhe onset of acute kidney injury (AKI) among patients who have survived cardiac arrest is not rare. Persistent acute kidney injury (PAKI) in the first 72 h after cardiac arrest, also occurs quite often [2,3]. About 30% of the CA hospitalized patients develop AKI syndrome after resuscitation [4], while the incidence of developing stage 3 AKI in patients with “out-of-hospital” cardiac arrest (OHCA) is 48.3% [5].

Evidence suggest that clinically significant AKI, is rarely associated with a single CA case followed by return of spontaneous circulation (ROSC), in the absence of postoperative respiratory complications (PRCs) [6]. The cases that have clinically significant AKI in the absence of PRCs are observed. Multiple organ dysfunction syndrome (MODS) follows the whole-body ischemia–reperfusion injury (WBIRI) and the post-resuscitation syndrome [7]. The abnormalities that are observed in post-resuscitation syndrome are similar to the abnormalities that occur in sepsis, as well as the abnormalities that are observed in immunity disorders [8]. In addition, clinically significant AKI after cardiac arrest is seen in patients with pre-existing renal impairment and in patients receiving RAAS (renin–angiotensin–aldosterone system) blockade therapy, a therapy that is used for the treatment of diabetic kidney disease (DKD) [9,10]. A recent experimental study in a CA swine model suggests that extracorporeal resuscitation by the administration of carbon monoxide (CO) may have therapeutic effects against renal failure, targeting the pro-apoptotic and the pro-inflammatory signaling pathways, suggesting a new possible treatment option against high mortality [11].

The renal ischemia–reperfusion model in cardiac arrest of the present study, describes the morphological and histological effects of ischemia in the right kidney, combined with the whole-body ischemia–reperfusion injury caused after cardiac arrest. Thus, the present study avoids the confusion with ischemic conditions resulting from surgical vascular manipulation, venous injury, or venous congestion. The experiment was based on swine, due to the size of these animals and their systemic circulation and renal function, which are quite similar to that of humans, compared to smaller experimental animals. The aim is to describe the histological and ultrastructural injuries observed in renal tissue after cardiac arrest.

## 2. Results

### 2.1. Light Microscopic Evaluation

**Figure 1:**
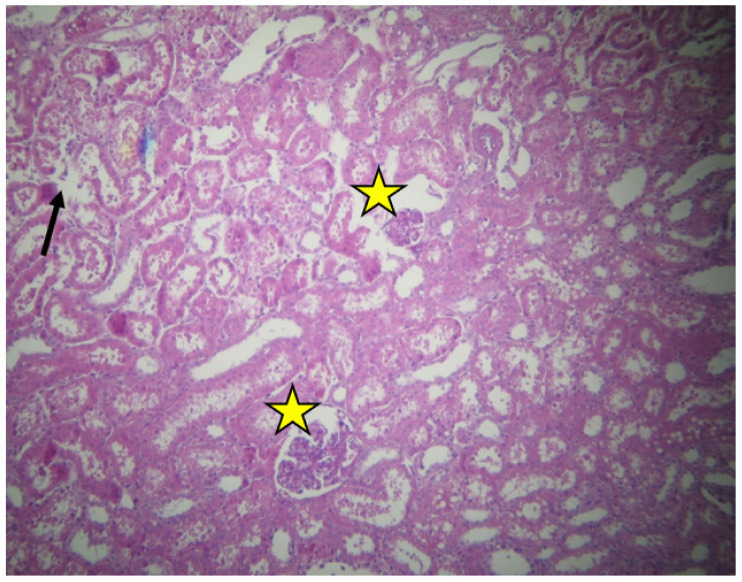
Cortex of non-resuscitated swine stained with HE. Renal corpuscles with abnormal urinary space (yellow stars). Destroyed tubular epithelium (black arrow), ×40.

No significant lesions were revealed in the tissue samples, apart from a few cases. The samples from the non-resuscitated swine group appeared to have more extensive damage. Renal corpuscles with enlarged and abnormal urinary spaces were detected (Figure 1).

**Figure 2:**
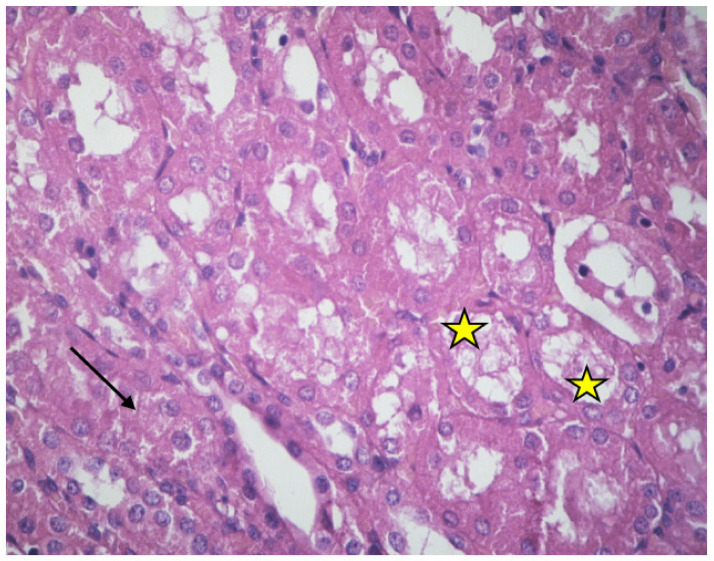
Cortex of non-resuscitated swine stained with HE. Destroyed tubular epithelium and amorphous material fills the tubular lumen (yellow stars). Expanded connective tissue (black arrow), ×160.

In some cases, the renal epithelial cells appeared to be damaged, while an amorphous material filled the renal lumen (Figure 2).

**Figure 3:**
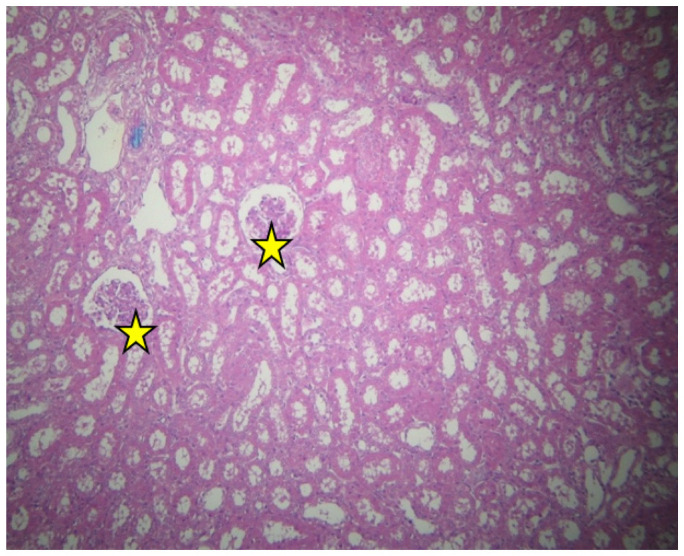
Cortex of resuscitated swine stained with HE. Normal renal corpuscles (yellow stars). Expanded connective tissue in few places, ×40.

There was also excessive connective tissue at several sites (Figure 2 and Figure 3).

**Figure 4:**
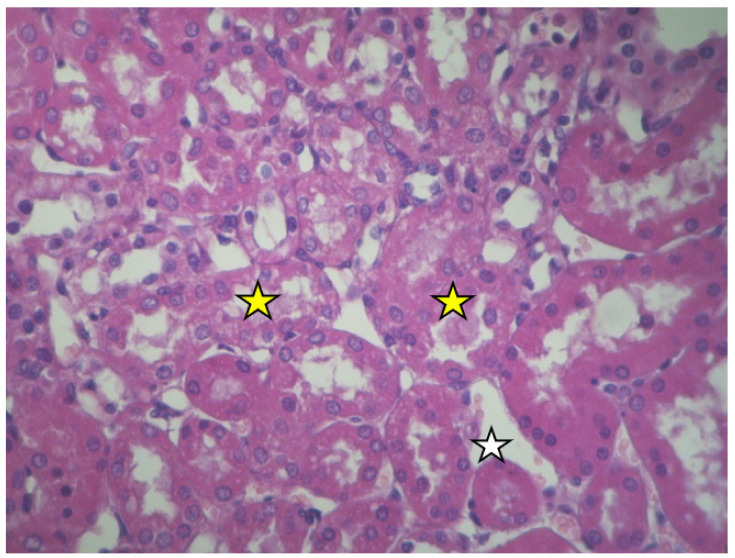
Cortex of resuscitated swine stained with HE. Destroyed tubular epithelium and amorphous material fills the tubular lumen (yellow stars). Expanded capillary (white star), ×160.

No injuries were detected in the kidney medulla. Some of the capillaries appear dilated (Figure 4).

### 2.2. Electron Microscopic Evaluation

**Figure 5:**
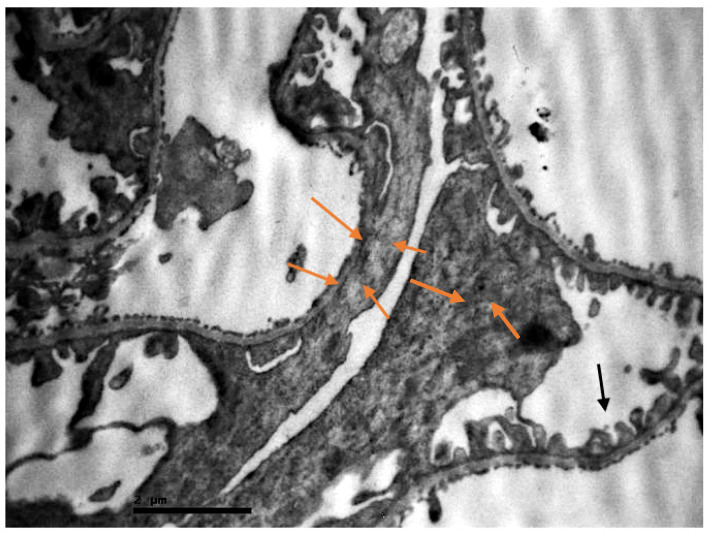
Renal corpuscle of resuscitated swine sample. Fusion of the podocytes (orange arrows). Relatively normal podocytes, not entirely separated as they should normally be (black arrow).

In the resuscitation samples, focal injuries of the renal parenchyma were observed. In the renal corpuscle, injuries were detected in a few loci, including enlargement of the urinary space and fusion of the podocytes (Figure 5).

**Figure 6:**
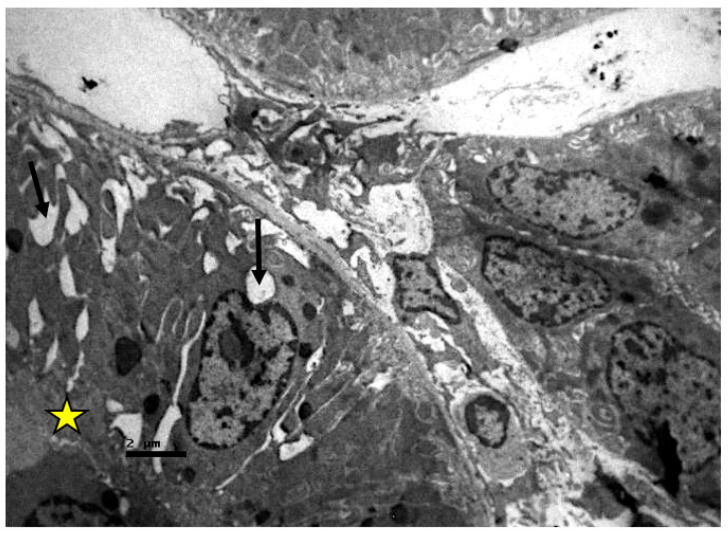
Proximal convoluted renal tubules of resuscitated swine sample. Vacuoles (black arrow) are observed in and between the epithelial cells, which also show absence of microvilli, on their surface (yellow star).

**Figure 7:**
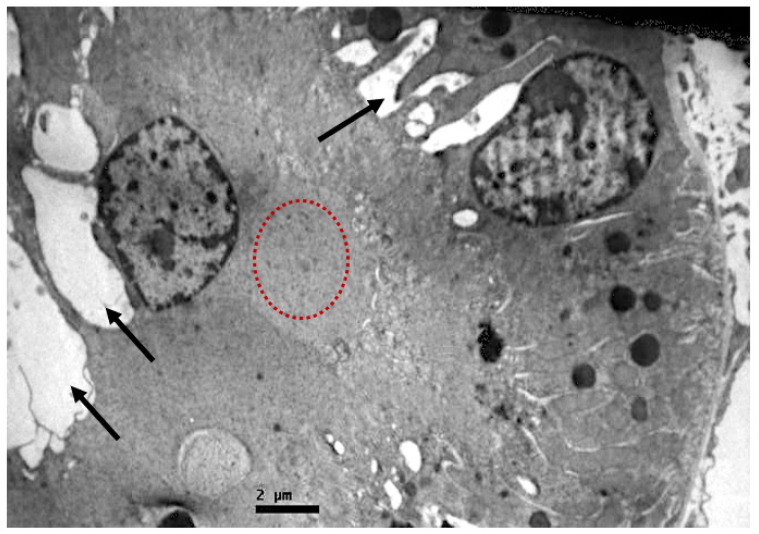
Proximal convoluted tubule epithelium of resuscitated swine sample. Cellular material is observed inside the lumen of the tubule (red dotted circle). Vacuoles (black arrows).

**Figure 8:**
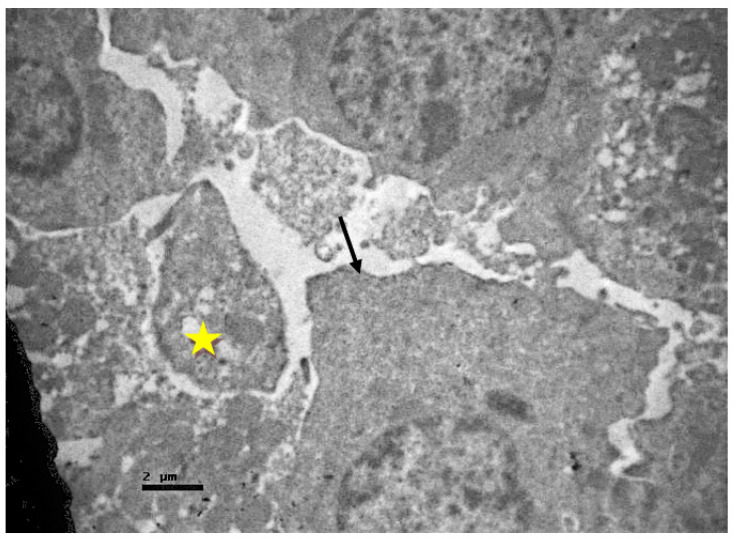
Cuboidal epithelial cells of proximal convoluted tubules of resuscitated swine sample. The cells appear signs of apoptosis (yellow star) and loss of surface microvilli (black arrow).

In the tubules, especially in the proximal ones, there was a reduction in the basal membrane invaginations, inter- and intracellular vacuolization, cellular apoptosis, and disorganization. Amorphous material was also detected inside their lumen (Figure 6, Figure 7 and Figure 8).

**Figure 9:**
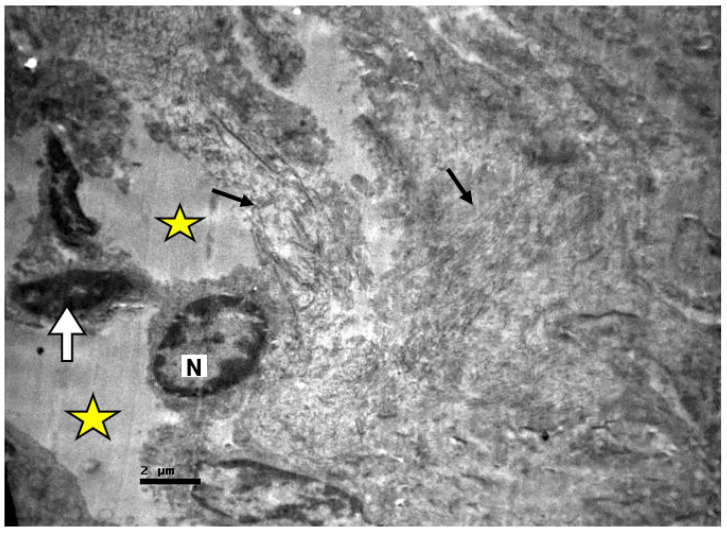
Connective tissue of resuscitated swine sample, that shows edema (yellow stars), activated fibroblasts (Ν), apoptotic cell (white arrow) and increased collagen fibers (black arrows).

**Figure 10:**
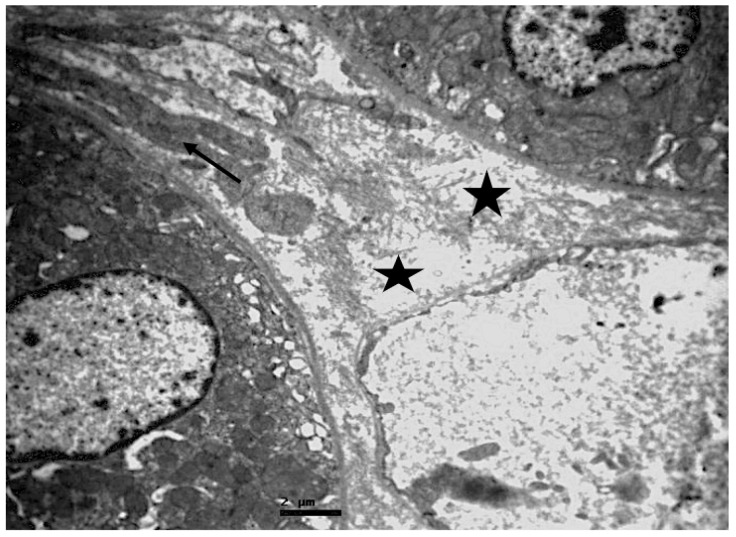
Connective tissue, of resuscitated swine sample that shows edema (black stars) and fibroblasts (black arrow).

Excessive collagen increase, fibrosis, and a plethora of apoptotic cells, mast cells, lymphocytes, mononuclear cells and congested capillaries were detected in the connective tissue. Prominent edema was also observed (Figure 9 and Figure 10).

**Figure 11:**
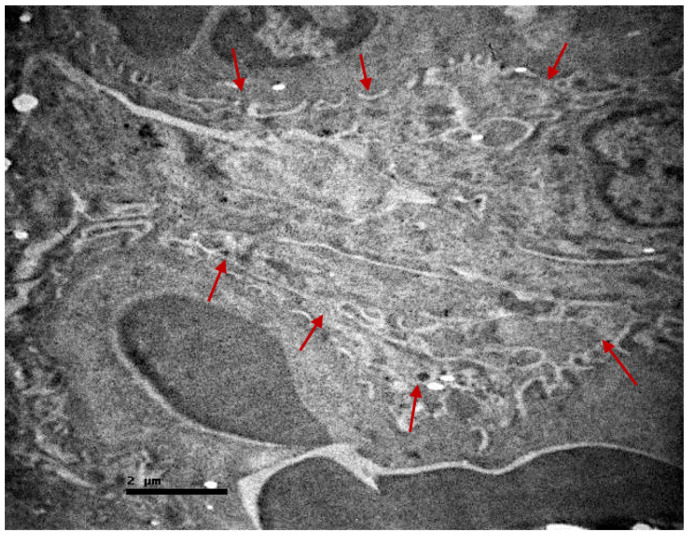
Renal corpuscle of non-resuscitated swine sample shows fusion of the podocytes (site among red arrows).

In the non-resuscitation samples, the injuries were more extensive. In the renal corpuscle, fusion of the podocytes was detected in many loci (Figure 11).

**Figure 12:**
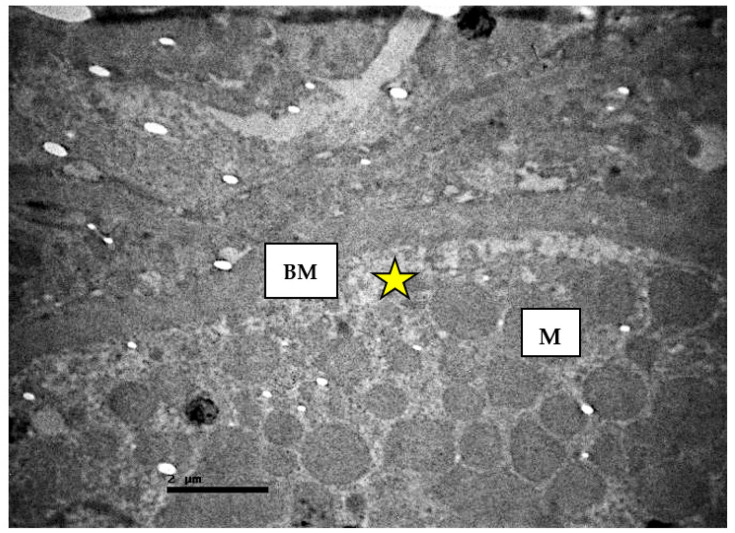
Renal convoluted tubules of non-resuscitated swine sample. The basement membrane of the epithelium shows thickness (BM) and disorganization of basal membrane invaginations (yellow star). Enlargement of the mitochondria (M) is also observed.

**Figure 13:**
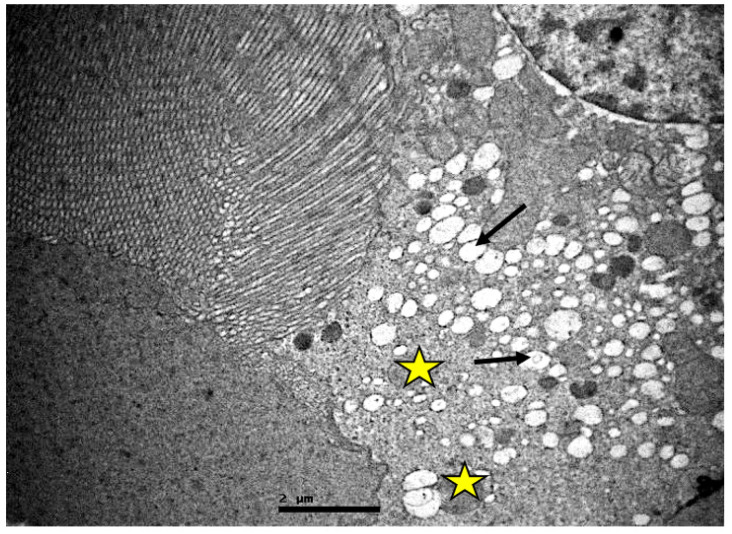
Renal convoluted tubules of non-resuscitated swine sample. Within the cell are identified large vacuolar spaces (black arrows) and swollen mitochondria (yellow stars).

In addition, destruction of the parietal layer cells and abnormal peripheral urinary space was observed. The renal tubules appeared disorganized and their microvilli were destroyed (Figure 12 and Figure 13). The distinguish amorphous material was detected again in the lumen of the tubules.

**Figure 14:**
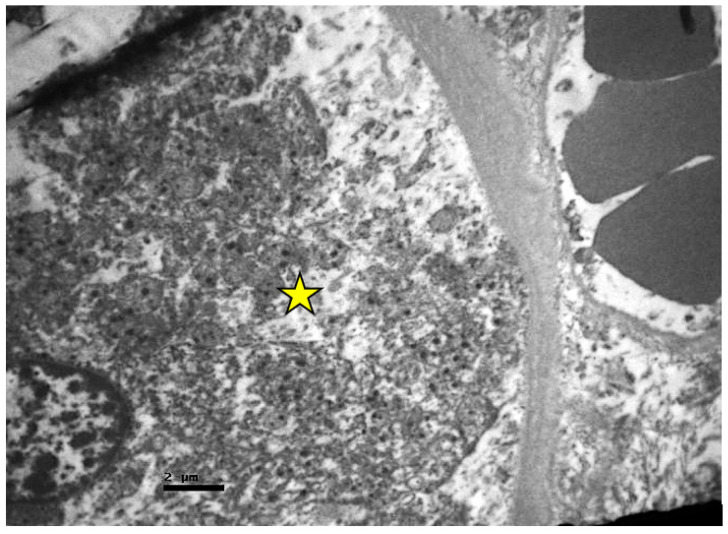
An apoptotic cell of the tubular epithelium of non-resuscitated swine sample (yellow star).

The basement membrane of the tubular epithelium was thickened. Moreover, the presence of intracellular vacuoles, enlarged mitochondria, and a plethora of apoptotic cells (Figure 14) were observed.

**Figure 15:**
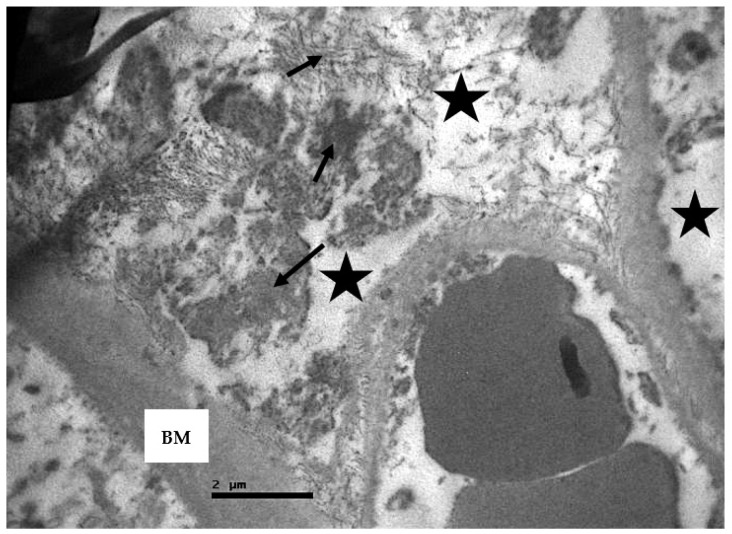
Connective tissue of non-resuscitated swine sample that showed fibrosis (black arrows) and edema (black stars). The basement membrane of the cuboidal epithelium appears thickened (BM).

**Figure 16:**
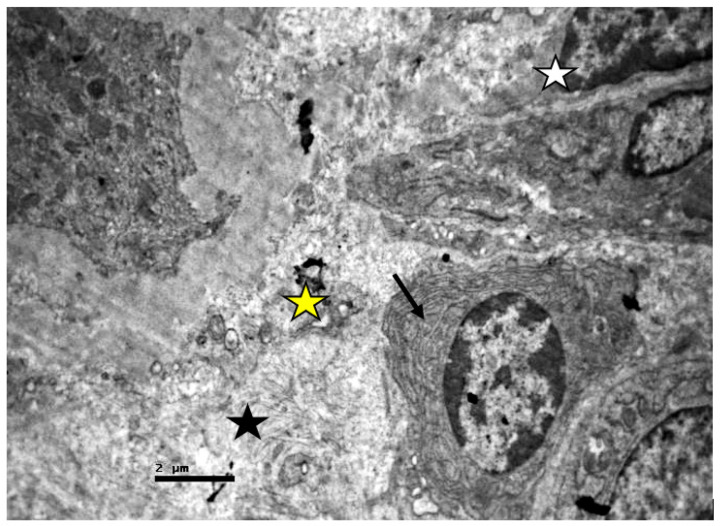
Connective tissue of no-resuscitated swine sample that shows apoptotic material (yellow star) and extensive edema (black star) and expanded capillary (white star), and active fibroblasts (black arrow).

The intervening connective tissue revealed the presence of edema, activated fibroblasts, fibrosis, macrophages, apoptotic material, and damaged vascular capillaries, some of which were full of erythrocytes (Figure 15 and Figure 16).

**Figure 17:**
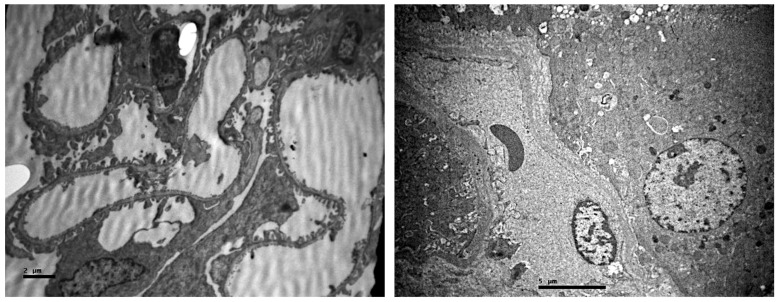
(**Left**) Normal renal corpuscle (2 μ). (**Right**) Normal renal connective tissue with normal capillaries. Normal renal tubules (5 μ).

Pictures of samples with no damage were also cited. These figures depicted the normal structure of the renal corpuscle and the normal renal connective tissue, in combination with normal renal tubules (Figure 17). The renal medulla seemed to be less affected than the renal cortex.

Two independent researchers assessed the extent of the damage in each sample. The following table represents a qualitative percentage and the descriptive extent of ultrastructural damage, evaluated by electron microscopy (Table 1).

## 3. Discussion

In cardiac arrest, renal microcirculation is disturbed, resulting in insufficient oxygenation of renal cells and the inability to metabolize cellular waste [12]. Hypoxia leads to the production of reactive oxygen species, nitric oxides, and reperfusion. It also triggers the inflammatory reaction, and the adhesion of leukocytes in the renal parenchyma, leading to the destruction of the proximal tubular epithelium and to AKI [13,14]. Due to the inability of renal cells to metabolize the cellular waste, a plethora of apoptotic cells were detected, with a significant decrease in the epithelial cells’ basal membrane invaginations, mainly in the cuboidal epithelium of the proximal tubules. In a rat model of cardiac arrest and cardiopulmonary resuscitation, histological damage of renal tissue, such as glomerular collapse, swelling of renal tubular epithelium cells, and infiltration of inflammatory cells were detected [15]. In addition to the glomerular collapse, there were abnormalities in the urinary space of the renal corpuscle and amorphous “material” in the urinary space. Evidence also suggests that post-resuscitation abnormalities after cardiac arrest are very similar to that of sepsis, in several organs [8]. Indeed, the lesions and injuries detected in the swine of the present study advocate for that suggestion. These findings agree with the literature regarding the incidence of AKI in the ROSC group [8,15].

The literature suggests that kidneys, after ischemia-repair processes, are infiltrated by a variety of immune cells, such as macrophages, mast cells, lymphocytes, and other mononuclear cells [16,17,18,19,20,21,22,23,24,25,26,27]. Macrophages infiltrate renal tissue in the early stages of repair, within the first 24 h after the reperfusion [16]. Activated macrophages exhibit strong phagocytic activity that should be further investigated in the future, alongside structural and ultrastructural morphological lesions [17,18]. Therapies based on leukocyte adhesion inhibitors appear to have a therapeutic effect against renal injury after ischemia [19,20]. Morphological research should also focus on the fact that T-cell-deficient nu/nu mice are significantly protected from renal injury after a whole-body ischemia–reperfusion injury (WBIRI) caused by cardiac arrest. This kind of protection is characterized by a decrease in serum creatinine SCr post-WBIRI and reduced tubular structural damage, which could be easily detected through transmission electron microscopy. A possible mechanism of protection involves a reduced ICAM-1 upregulation post-WBIRI in T-cell-deficient mice [21]. In addition, a large number of studies in the isolated clamp model of renal ischemia–reperfusion injury, (IRI), indicates T lymphocytes, and especially CD4 + T lymphocytes, as one of the major mediators in causing acute renal failure (ARF) [22,23,24,25]. Finally, another interesting biomarker with clinical significance relating to injured renal tubules (which can be morphologically detected) is the neutrophil gelatinase-associated lipocalin [26].

A severe acute inflammatory reaction eventually leads to renal tissue fibrosis and AKI [27,28,29]. The damage appeared more severe in swine to which the return of spontaneous circulation was not achieved. This observation is confirmed by epidemiological data which supports that the severe kidney damage and persistent AKI in the first 72 h after cardiac arrest (PAKI) appears to be related to the severity of the disease and the patient’s health outcome [1,2,5,30].

Recent studies suggest that Toll-like receptor 4 (TLR4) mediates an inflammatory response in AKI [31]. Research focuses on TLR4 as a therapeutic intervention for AKI and similar renal injuries. In addition, levosimendan has been proposed for dealing with AKI by improving possible mitochondrial dysfunction and reducing the mitochondrial apoptosis [32]. Indeed, mitochondrial dysfunction and apoptosis were detected on several specimens in the present study.

## 4. Materials and Methods

The experimental protocol was approved by the Department of Animal Care and Use Committee of the Greek Ministry of Agriculture, according to the European Community Guiding Principles for the Care and Use of Animals (EU Directive 2010/63/EU, Protocol No. 110942/756/2015). The experiment was conducted in the AHEPA Hospital of the Aristotle University of Thessaloniki, Greece. The protocol included 24 healthy female Munich swine of three months old and approximately 23.0 ± 2.9 kg of body weight. The animals were premedicated, intubated and mechanically ventilated [3,33,34]. None of the animals was receiving any medication before the experiment. Female swine were chosen because the catheterization of their urinary bladder is easier. The femoral veins and the femoral artery were cannulated. In order to be hemodynamically monitored, a 7.5Fr Swan-Ganz CCOmboV catheter was inserted into the right femoral artery of each animal through an 8Fr sheath. Cardiac arrest was induced via a pacemaker wire placed through the sheath of the Swan-Ganz catheter.

Seven minutes later and without any interventions, cardiopulmonary resuscitation (CPR) was performed, using the LUCAS CPR device and FIO_2_ 21% for five minutes. Afterwards, advanced life support (ALS) was applied with rate analysis, defibrillation and/or drug administration, based on the European Resuscitation Council (ERC) Guidelines [35,36].

The above process resulted in the main groups: animals to whom the return of spontaneous circulation was achieved (ROSC; *n* = 10) in a median of 10 min, (10 swine resuscitated at 18, 17, 7, 7, 12, 17, 6, 10, 8, and 10 min), and those to whom the return was not achieved after 40 min of CPR (no ROSC, *n* = 14).

To the no ROSC animals, CPR was terminated and tissue samples were obtained. The ROSC animals were supported for 4 h; they remained mechanically ventilated and hydrated, as before the cardiac arrest induction, with no further need for other treatment. After 4 h they were euthanatized with thiopental and potassium chloride administration; tissue samples were obtained from both groups. The samples were surgically removed from the lower pole of the right kidney of each swine. Kidney tissue samples to 3 mm thick, 20 mm × 30 mm were obtained by the use of a scalpel. The samples included the cortex and medulla from each kidney. Specimens were prepared for electron microscopy examination, after being sectioned into <1 mm^3^ pieces.

### 4.1. Transmission Light Microscopy

After the collection of tissues, samples were placed in a formol solution (10% formaldehyde) for 24 h. Then, samples were processed through the Histokinette (MYR STP 120-1, Spain) for 14 h, for fixation, dehydration, and clearing. These processes were followed by the paraffin infiltration at 60 °C. The tissue-paraffin blocks were microtomed into three micrometer-thickness (3 μ) tissue sections. Some of these sections were stained with Hematoxylin and Eosin, EH in order to be used for light microscopic evaluation [3].

### 4.2. Transmission Electron Microscopy (TEM)

Sectioning into <1 mm^3^ pieces was applied to the kidney tissue samples. Kidney tissue samples were then submerged into 3% glutaraldehyde for 2 h, followed by 1 h in 1% osmium tetroxide (OsO_4_). Then, staining was carried out with 1% uranyl acetate for 16 h, followed by dehydration with high ethanol concentrations. Finally, the specimens were embedded into Epon resin, and ultra-thin sections (60–90 nm) were taken, which were then stained with Reynold’s stain. Lastly, the renal tissue samples were examined in a TEM JEOL 1011 at 80 kV (JEOL-Tokyo, Tokyo, Japan).

## 5. Conclusions

The kidneys have a strong ability for self-repair and their normal function usually returns after the recovery of the patient. Although AKI is not the leading cause of death after cardiac arrest, cardiac arrest causes damages to kidney tissue, which can affect the prognosis of the patient’s health outcome, depending on the extent, severity, and persistence of this damage. Also, depending on the severity and co-morbidities of each clinical case, there is a high probability that AKI could evolve to chronic kidney disease [37]. Renal injuries after cardiac arrest contribute to multiple organ dysfunction syndrome (MODS) and they can lead to the patient needing hemodialysis and even to death, after resuscitation. The present study revealed histological and ultrastructural lesions on the kidneys after cardiac arrest. This damage was more conspicuous in the no ROSC group. However, there is a need for further studies to determine to what extent these injuries affect the patient’s health after a cardiac arrest and to compose therapeutic ways to reverse the adverse outcomes.

## Figures and Tables

**Table 1:** Electron microscopy qualitative percentage of ultrastructural damage and lesions, as observed in both groups.

Extent of Damage	ROSC% ^1^ (*n* = 10)	No ROSC% ^1^ (*n* = 14)
No damage detected	20% (2)	14% (2)
Mild damage detected	50% (5)	29% (4)
Moderate damage detected	30% (3)	36% (5)
Extensive damage	0% (0)	21% (3)

^1^ Return of Spontaneous Circulation.
